# Protoscolicidal effects of curcumin nanoemulsion against protoscoleces of *Echinococcus granulosus*

**DOI:** 10.1186/s12906-023-03927-8

**Published:** 2023-04-18

**Authors:** Aref Teimouri, Sanaz Jafarpour Azami, Saeedeh Hashemi Hafshejani, Mohammad Ghanimatdan, Mohammad Saleh Bahreini, Rasoul Alimi, Seyed Mahmoud Sadjjadi

**Affiliations:** 1 Department of Parasitology and Mycology, School of Medicine, Shiraz University of Medical Sciences, Shiraz, Iran; 2 Department of Medical Parasitology and Mycology, School of Public Health, Tehran University of Medical Sciences, Tehran, Iran; 3 Department of Epidemiology and Biostatistics, School of Health, Torbat Heydariyeh University of Medical Sciences, Torbat Heydariyeh, Iran

**Keywords:** Curcumin nanoemulsion, Protoscolicidal, *Echinococcus granulosus sensu stricto*

## Abstract

**Background:**

The aim of the present study was to assess in vitro protoscolicidal effects of curcumin nanoemulsion (CUR-NE) against protoscoleces of cystic echinococcosis (CE)/hydatid cysts.

**Methods:**

The CUR-NE was prepared via spontaneous emulsification of soybean as the oil phase, a mixture of Tween 80 and Tween 85 as the surfactant, ethanol as the co-surfactant and distilled water. Various concentrations of CUR-NE (156, 312, 625 and 1250 µg/ml) were exposed to collected protoscoleces of infected sheep liver hydatid cysts for 10, 20, 30, 60 and 120 min. Viability of the protoscoleces were assessed using eosin exclusion test. Morphological changes of the protoscoleces were observed using differential interference contrast (DIC) microscopy.

**Results:**

The mean particle size and zeta potential of CUR-NE included 60.4 ± 14.8 nm and − 16.1 ± 1.1 mV, respectively. Results showed that the viability of the protoscoleces decreased significantly with increases in CUR-NE concentrations (*p* < 0.001). The mortality rates of protoscoleces with exposure to concentrations of 1250 and 625 µg/ml of CUR-NE for 60 min were 94 and 73.33%, respectively. Mortality of the protoscoleces was 100% after 120 min of exposure to 1250 and 625 µg/ml concentrations of CUR-NE. Using NIC microscopy, extensively altered tegumental surface protoscoleces was observed after protoscoleces exposure to CUR-NE.

**Conclusion:**

The findings of the present study revealed the in vitro protoscolicidal potential of CUR-NE. Therefore, CUR-NEs are addressed as novel protoscolicidal agents, which can be used as an alternative natural medicine to kill the protoscoleces, owing to their low toxicity and significant inhibition potency. However, further studies are necessary to investigate pharmacologic and pharmacokinetics of CUR-NEs.

## Introduction

Cystic echinococcosis (CE)/hydatidosis is a cosmopolitan zoonotic tapeworm disease with various clinical complications in humans and herbivores. It is caused by the larval stage of *Echinococcus granulosus* sensu lato [1]. Based on the World Health Organization (WHO) reports, CE is one of the 17 neglected tropical diseases (NTDs) with an important challenge from medical and economic points of view [2]. The CE causative agents are mostly transmitted between the canines (primary definitive hosts) and various livestock species (intermediate hosts). Human and other intermediate hosts become infected through accidental direct ingestion of the infective eggs of *E. granulosus* sensu lato as well as contaminated water and/or foods [3–6]. The disease usually develops in the host liver and lungs with less rate in other organs, including brain and bones of the intermediate hosts [1]. Clinical characteristics of CE depend on various factors, including involved organs, locations, numbers and sizes of cysts in the involved organs as well as mass effects within the organs and the surrounding structures [5]. Currently, various single or combined options for the treatment of CE are available, including surgery, percutaneous methods [puncture, aspiration, injection and re-aspiration (PAIR)] and chemotherapy with benzimidazole derivatives (mebendazole and albendazole) for live cysts, as well as “watch and wait” method for the silent cysts based on image classifications following stage-specific approaches [7]. Toxic side effects with high frequencies are the major limitations of chemical drugs. The frequent side effects are alopecia, hepatotoxicity, leucopenia, osteoporosis, teratogenicity and thrombocytopenia [8–11]. Additionally, the most important complications of surgery and PAIR for the treatment of CE include possible ruptures of the cysts or leakage of the cyst protoscoleces contents that can be lead to anaphylactic shock, secondary infections, and even death of the patients [12, 13]. To solve these problems, before surgery, the surgeons usually use a broad spectrum of protoscolicidal agents such as 20% hypertonic saline, silver nitrate and cetrimide to decrease risks of spillage of viable protoscoleces and possible recurrence episodes [5]. However, serious complications such as biliary fibrosis, hepatic necrosis, and cirrhosis have limited use of these agents [5]. Therefore, the use of novel protoscolicidal substances for intraoperative killing of protoscoleces with high efficacy and low side effects are necessary during surgery.

Curcumin (CUR) [1,7-bis(4-hydroxy-3-ethoxyphenyl)-1,6-heptadien-3,5-dione] is a natural phenolic compound extracted from the ground rhizomes of a perennial herb, *Curcuma longa* Linnaeus. Pharmacological safety and efficacy of CUR make it a potential compound for the treatment and prevention of various diseases such as chronic diseases, allergies, arthritis, wounds, metal-induced liver damage, diabetes, migraine, Alzheimer’s disease and neurological disorders [14–19]. In addition to its harmless nature, investigations on pharmacological properties of CUR have shown extensive ranges of promising biological and pharmacological activities, including anti-microbial [20], anti-inflammatory [21], anti-osteoarthritis [22] and anticarcinogenic properties [23]. Furthermore, cytotoxic and parasiticidal issues of CUR have been demonstrated in helminthic parasites such as *Schistosoma mansoni*, *S. japonicum* [24] and a wide range of protozoan parasites such as *Leishmania spp.* [25, 26], *Giardia lamblia* [27], *Trypanosoma* [28, 29], *Plasmodium falciparum* [30] and *Toxoplasma gondii* [31]. Morover, the in vitro efficacy of chitosan-curcumin [32] and chitosan nanoparticles (NPs) [33] have been evaluated against protoscoleces of *E. granulosus.*

Despite numerous advantages of CUR, hydrophobic nature of the CUR derivatives, low aqueous solubility, chemical instability, poor bioavailability, short half-life and rapid metabolism create serious challenges to its effectiveness [34]. In recent years, nanotechnology has been introduced to medical societies as an advanced technology for addressing these limitations [34]. Since it can increase the solubility and cellular uptake efficiency, dissolution, and bioavailability of materials at the desired site of action and, consequently, improve the therapeutic effectiveness [35, 36]. Moreover, the nanomaterials can improve cell penetration and also maintain effective intracellular delivery and accumulation [35]. Various kinds of formulations have been used to tackle this issue. From these formulations, nanoemulsion (NE) has been technologically advanced to improve CUR solubility and bioavailability [35]. These NEs or fine oil-in-water dispersions stabilized with small quantities of emulsifiers are very small and hence cannot scatter the light beams. Thus, NEs seem clear despite their opaque appearance [37]. Despite the studies on natural products such as *Curcuma* extracts and its derivatives against protoscoleces of CE [38–40], no study has been carried out on the effects of curcumin nanoemulsion (CUR-NE) on protoscoleces of CE, so far. Therefore, the aim of this study is in vitro assessment of the protoscolicidal efficacy of CUR-NE against protoscoleces of CE.

## Materials and methods

### Compounds

CUR (CAS-No:458-37-7; Sigma-Aldrich; Purity: ≥80%), Soybean oil and eosin powder (Sigma-Aldrich) were used for the study. In addition, sodium chloride, ethanol, methanol and polysorbates of Tween 80 and Tween 85 (Merck, Germany) were also used in this work.

### Preparation of curcumin nanoemulsion

The CUR-NE was successfully prepared as described previously by the authors using spontaneous emulsification of soybean as the oil phase, a mixture of Tween 80 and Tween 85 as the surfactant, ethanol as the co-surfactant and distilled water. Prepared CUR-NE was characterized using Malvern Zetasizer Nano ZS instrument and transmission electron microscopy [31].

### Protoscoleces collection

Protoscoleces of *E. granulosus* sensu stricto were collected from livers of the naturally infected sheep, slaughtered at Shiraz slaughterhouse, Fars Province, Southern Iran. Protocols for the preparation of protoscoleces and viability assessment were previously described by Sadjjadi et al. [41].

### In vitro protoscolicidal activity

In the current study, a concentration of 1250 µg/ml of CUR-NE was prepared, solution then being a 1/2 dilution in the series. Various concentrations of CUR-NE, including 1250, 625, 312 and 156 µg/ml, were used for various exposure times, including 10, 20, 30, 60 and 120 min. Initially, 0.5 ml of the protoscoleces (2 × 10^3^/ml) solution was transferred into test tubes. Then, 0.5 ml of various CUR-NE concentrations were added to each test tube and mixed well. Test tubes were incubated at 37 °C for 10, 20, 30, 60 and 120 min. Then, the upper phase of the mixture was removed carefully and 25 µl of eosin stain (0.1%) was added to the pellet of protoscoleces and gently mixed. Protoscoleces sediment were smeared on glass slides. Using light microscopy the viability protoscoleces were measured [42]. Phosphate buffered saline (PBS) solutions containing Tween 80 (10%) as surfactant in the preparation of CUR-NE was used as negative control. A solution of 20% hypertonic saline was also used as positive control. Experiments were carried out in triplicate and the mean and standard deviation (SD) were calculated for the samples.

### Viability assay

**Fig. 1:**
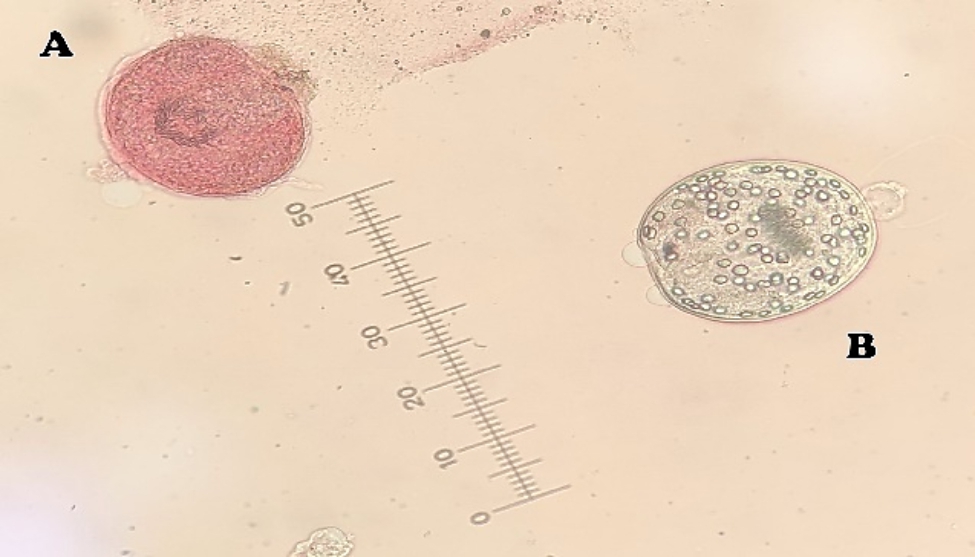
Dead (**A**) and live (**B**) protoscoleces after staining with 0.1% eosin

The viability of the protoscoleces was evaluated by their flame cell motility under light microscope as well as impermeability to 0.1% eosin solution. Technically, dead protoscoleces absorb eosin and become red and live protoscoleces do not absorb eosin (no color), demonstrating typical muscular movements and flame cell activity (Fig. 1). Moreover, mortality rate of the protoscoleces was calculated and reported as the proportion of dead protoscoleces to total protoscoleces [43].

### Assessment of the morphologic structure of protoscoleces using differential interference contrast (DIC)/Nomarski microscopy

The morphological changes of protoscoleces including calcareous corpuscles structure changes, disorganization of the hooks and destruction of tegument were carefully observed in control and treated (CUR-NEs) groups using DIC/Nomarski microscopy.

### Statistical analysis

Statistical analysis was conducted using GraphPad Prism Software v.8.0.0 for Windows (GraphPad, USA). Statistical differences between the treatment and control groups were reported using one-way analysis of variance (ANOVA) with 95% confidence intervals (CI) followed by Games-Howell multiple comparisons test [44]. Results were recorded as mean ± SD and *p*-values < 0.05 were reported as significant.

## Results

### Characterization of curcumin nanoemulsion

**Fig. 2:**
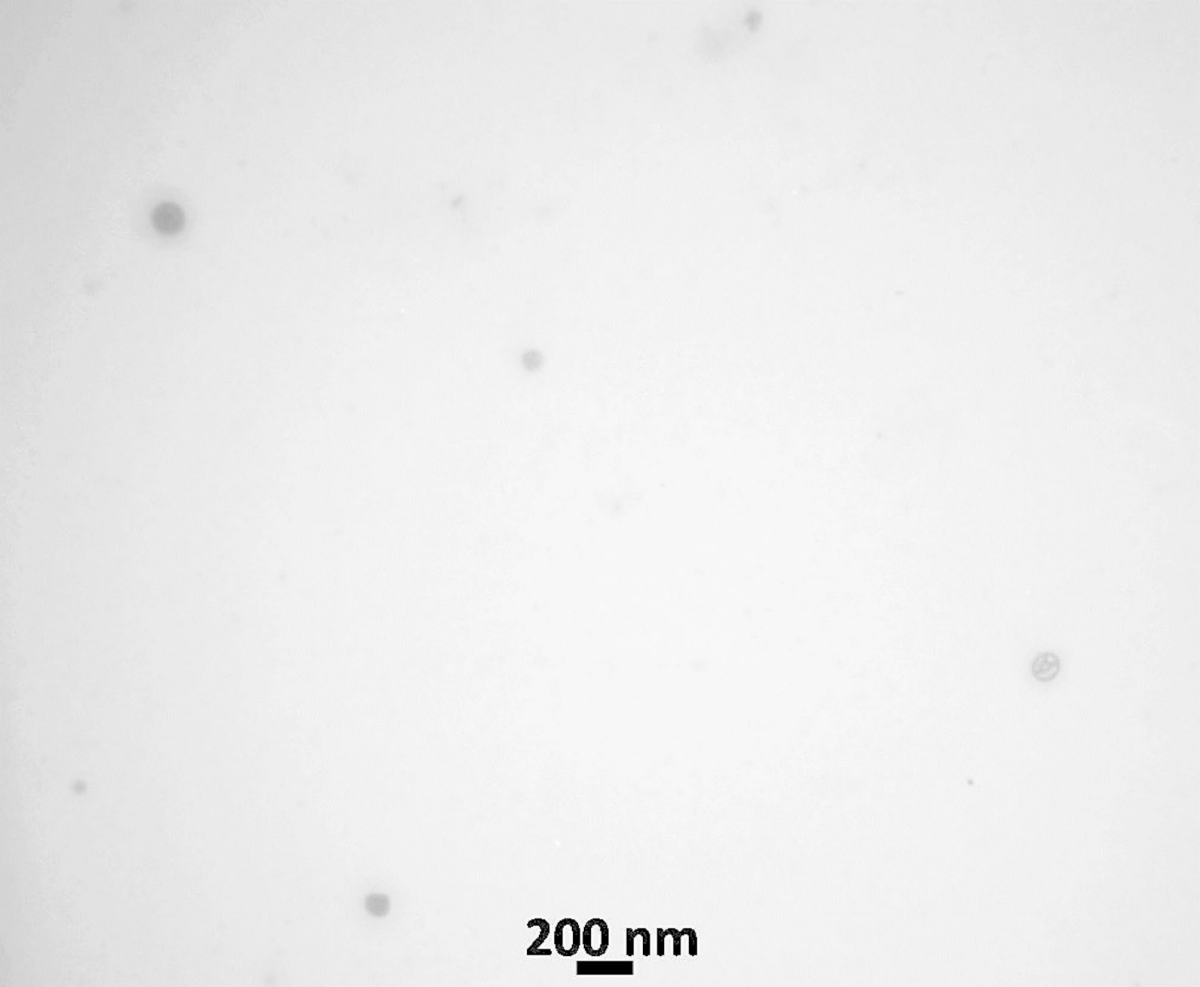
Transmission electron micrograph of the curcumin nanoemulsion

In this study, CUR-NEs were successfully synthesized. The mean particle size of CUR-NEs by DLS was 60.4 ± 14.8 nm and their zeta potential was − 16.1 ± 1.1 mV. The spherical shape and size of the CUR-NEs were verified using TEM (Fig. 2).

### In vitro experiments

**Fig. 3:**
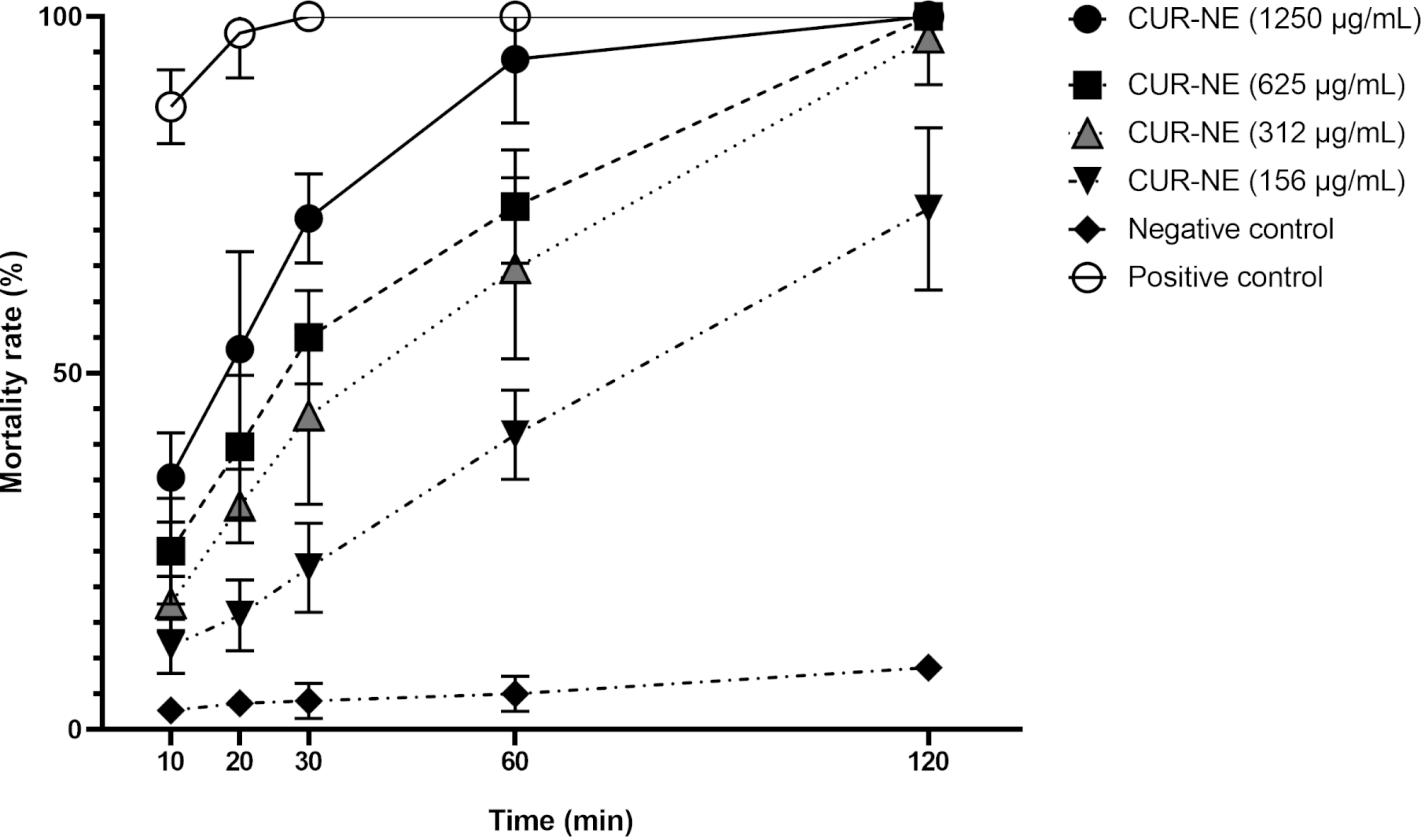
In vitro protoscolicidal effects of curcumin nanoemulsion against protoscoleces of *Echinococcus granulosus* sensu stricto at various concentrations and exposure times. Positive control, 20% hypertonic saline; negative control, phosphate buffered saline containing Tween 80

In the present study, the viable protoscoleces upper than 90% were used for further experiments. Viable protoscoleces remain colorless and show amoeboid like movement and flame cell activity under light microscope. Simultaneously, non-viable protoscoleces get colored by eosin staining. Figure 3 shows in vitro protoscolicidal activity of various CUR-NE concentrations (156, 312, 625 and 1250 µg/ml) with various exposure times (10, 20, 30, 60 and 120 min) against protoscoleces of CE. Statistically, differences between the protoscolicidal effects of CUR-NEs were significant for all concentrations and exposure times, compared with the negative control (*p* < 0.001). Results showed that viability of the protoscoleces decreased significantly with increases in CUR-NE concentrations (*p* < 0.001). At concentrations of 1250 and 625 µg/ml for 60 min, mortality rates were measured as 94% and 73.33%, respectively. A 100% mortality rate was observed at 1250 and 625 µg/ml concentrations of CUR-NEe after 120 min. The minimum protoscolicidal activity of CUR-NEs included 11.67% (156 µg/ml, 10 min). ANOVA analysis demonstrated statistically significant differences between the mean rates of protoscoleces mortality in treatment groups at various exposure times, compared with the negative control (*p* < 0.001).

### Morphological and structural analyses of treated protoscoleces of CE

**Fig. 4:**
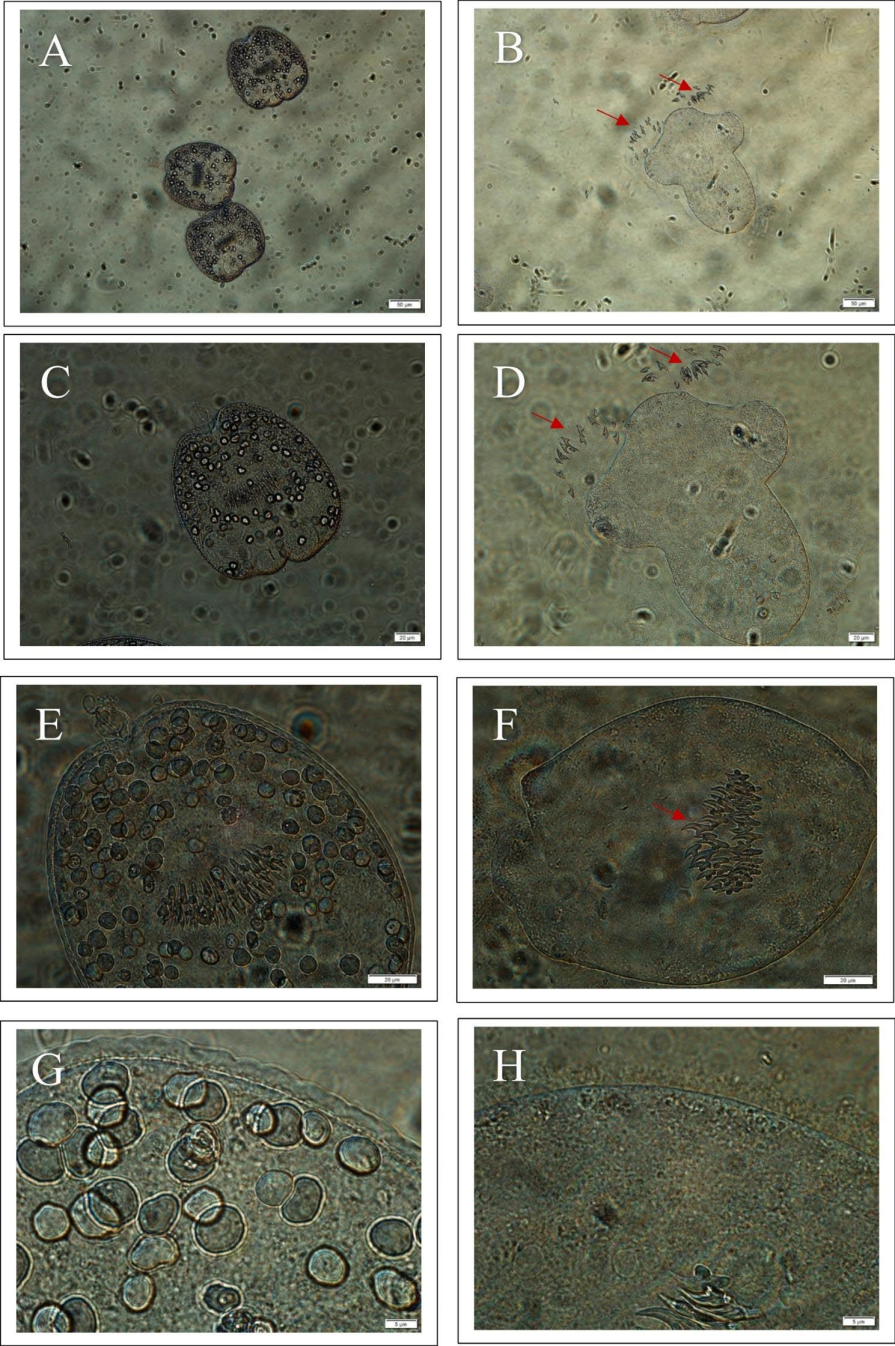
**A**, **C**, **E** and **G**, micrographs of the hydatid cyst protoscoleces of the control group, showing intact, stable tegument uniformed within the entire cell perimeter as well as obvious calcareous corpuscles and normal arrangement of hooks on the rostellum; **B**, **D**, **F** and **H**, micrographs of the hydatid cyst protoscoleces in groups treated with 1250 µg/ml of CUR-NEs, showing thin and tear tegument (3 H), faded calcareous corpuscles and disorganization of rostellar hooks, Notes: red arrow in **B**, **D**, and **F** micrographs points at hooks

Normal morphology was observed in untreated (control) protoscoleces of CE with intact, stable tegument uniformed within the entire cell perimeter and obvious calcareous corpuscles as well as normal arrangement of hooks on the rostellum (Fig. 4A, C, E and G). In contrast, the treated protoscoleces showed loss of viability, and morphological changes such as severe damage to the tegument, reduction in the size and number of the calcareous corpuscles and disorganization of rostellar hooks (Fig. 4B, D, F and H).

## Discussion

Natural medicines have been used for different parasitic diseases for centuries. Due to the good biodegradability and safe nature for the host organs, traditional and natural medicines have been studied extensively for drug discoveries in recent decades [45–48]. Nowadays, several available drugs originate from herbal sources and some of the effective drugs are naturally based [49]. CUR is a natural, nontoxic polyphenolic phytochemical, which has been used for several centuries as a therapeutic and health-promoting agent [20]. After the first scientific report by Oppenheimer (1937) on the use of CUR in human biliary disease [50], interests in studies on CUR have increased dramatically. Safety, tolerability and nontoxicity of CUR are well-verified in animals and humans even at high doses [51–54]. CUR and its derivatives have extensively been popular due to their anti-inflammatory [21, 53] and anti-microbial effects [55, 56]. Up-to-date studies have been carried out to assess effects of *Curcuma* extract and its derivatives against protoscoleces of CE [38, 40]. More recently, efficacy of *C. longa* essential oil (CLEO) against protoscoleces of CE was assessed and demonstrated that protoscoleces were completely killed after 5 and 10 min of exposure to doses of 200 and 100 µl /ml CLEO, respectively [40]. Similar studies showed that ethanolic extract of *C. longa* includes antiparasitic effects against protoscoleces of CE and the mortality rates of protoscoleces following exposure to *C. longa* extract at 50 mg/ml were 71.0, 81.3 and 93.2% after 10, 20 and 30 min, respectively [38].

Similar to the natural form of CUR, its nanoform is able to prevent and eliminate the growth of various microbes such as bacteria, fungi and parasites. The present study showed protoscolicidal effects of CUR-NE against protoscoleces of CE. Results demonstrated that viability of protoscoleces decreased significantly with increases in all concentrations of CUR-NEs (*p* < 0.001). The current findings showed that CUR-NEs included potent protoscolicidal activities, especially after 120 min at 1250 and 625 µg/ml (100% mortality rate). Similarly, Azami et al. (2018) showed potential of CUR-NEs in treatment of acute and chronic toxoplasmosis in mouse models [31]. Naturally, NEs provide larger surfaces, including potency of increased solubility. This potency is mostly due to the large interfacial adsorption of the core compounds, increased bioavailability due to the fast delivery of the active compounds to plasma membranes (PM) and organized releases of the drugs [57]. Furthermore, NPs such as silver, chitosan and CUR have been used for the treatment of giardiasis in experimental animal models, and results showed that the number of the parasites in stool and small intestinal sectors decreased in treated rats, compared with non-treated ones [58]. In a recent study, mortality rates of protoscoleces respectively included 28 and 32% after 60 min of exposure to 4 mg/ml chitosan and CUR, while the mortality rate was nearly 68% in the presence of chitosan NPs containing CUR (Ch-Cu NPs) at a same concentration after 60 min [59]. Although, pharmacological mechanisms of CUR are possibly associated with the compound inhibition of various biological cell signaling pathways and enzymes, the exact molecular mechanisms of CUR parasiticidal activity need further investigations [59].

In recent years, multiple inorganic NPs have been assessed against protoscoleces of CE [59, 60]. Mahmoudvand et al. (2014) showed that biogenic selenium NPs (Se-NPs) at all concentrations have potential protoscolicidal effects against protoscoleces of CE [61]. Rahimi et al. revealed protoscolicidal activity of the green synthesized silver NPs (Ag-NPs) and reported 90% mortality rate of protoscoleces using 0.15 mg/ml Ag-NP [62]. Nematollahi et al. compared protoscolicidal effects of Se-NPs and Ag-NPs and reported that the Se-NPs included higher protoscolicidal effects than those the Ag-NPs did [63]. Napooni et al. reported that gold NPs (Au-NPs) at 4 mg/ml concentrations killed 76% of the protoscoleces within 60 min [64]. More recently, Ezzatkhah et al. (2021) reported potent protoscolicidal effects of Copper-NPs, especially in combination with albendazole, which entirely eliminated the parasites after 10 − 20 min of exposure [65]. It has been verified that nanomaterials can interact with various living molecules and microbes because of their large surface-to-volume ratio and easier entry into the cells, compared to other particles. Therefore, nanomaterials can interrupt microbial pathogens, especially parasites [66].

Based on the several studies, the mean size of curcuminoid NEs is often less than 100 nm [67]. In the current study, the average particle size and zeta potential of the prepared CUR-NE included 60.4 ± 14.8 nm and − 16.3 ± 1.1 mV, respectively. Additionally, high stability of the present CUR-NEs was seen during 2 months of storage at room temperature. This can be associated to highly negative zeta potential of the present CUR-NEs. Highly positive and negative zeta potentials have been demonstrated in experiments to technically serve stabilities of microemulsions (MEs) and NEs because of their highly charged surfaces that resist aggregation of droplets [68]. Usually, conventional emulsions include low stabilities as shown by sedimentation of stored CUR at RT. Often, NEs lead to an improved physical stability [69]. However, CUR does not come to contact with water in the external phase because CUR is absorbed into the oily phase. Hence, NEs seem to provide inactive conditions for CUR. In NEs, CUR is actively protected from degradation [69].

In the current study, more detail and more clear observation of morphological changes of protoscoleces were observed using DIC microscopy [70]. Antibacterial mechanisms of CUR are well documented [71]. CUR uses multiple mechanisms to kill *Candida albicans*, including signaling alteration, cell wall integrity loss, metabolic shift, cell stress, DNA synthesis and repair, hyphal development, mitochondrial integrity and transcriptional and translational regulation. Of these mechanisms, CUR significantly affects genes that control cell wall integrity, because most of the genes of the pathway were downregulated [71]. Alterations in cell membrane permeability reveal changes in the physical state of the membrane or compromised cell wall integrity. These changes can result in leakages of proteinaceous constituents and other cell contents. However, the modes of action of CUR in parasitic diseases are not clearly understood and further studies are necessary to precisely investigate the exact action mechanisms of CUR on parasites, especially protoscoleces of CE.

## Conclusion

This is the first report on in vitro effects of CUR-NEs, as protoscolicidal agents, on protoscoleces of *E. granulosus* sensu stricto. The results showed effective, promising protoscolicidal activities. Therefore, CUR-NEs are addressed as a novel protoscolicidal agents, which can be used as alternative natural medicine to kill the protoscoleces, owing to their low toxicity and significant inhibition potency. However, the use of CUR-NE therapeutics is still in its primary stage. Further studies are necessary to investigate the pharmacologic and pharmacokinetics of CUR-NEs.

## Data Availability

All data generated or analyzed during this study are included in this published article. The raw data are available from the corresponding author upon reasonable request.
